# Changes in the Quality of Life and Nutrition Markers in Transition From End-Stage Kidney Disease to Kidney Transplantation: Insights From a Vietnamese Cohort

**DOI:** 10.7759/cureus.62105

**Published:** 2024-06-10

**Authors:** Thu-Ha Nguyen, Rozita Mohd, Zulfitri 'Azuan Mat Daud, Ruzita Abd Talib, Bee-Koon Poh

**Affiliations:** 1 Center for Community Health Studies, Universiti Kebangsaan Malaysia Faculty of Health Sciences, Kuala Lumpur, MYS; 2 Department of Nutrition, 108 Military Central Hospital, Hanoi, VNM; 3 Unit of Nephrology, Department of Medicine, Universiti Kebangsaan Malaysia Faculty of Medicine, Kuala Lumpur, MYS; 4 Department of Dietetics, Universiti Putra Malaysia Faculty of Medicine and Health Sciences, Serdang, MYS

**Keywords:** health-related quality of life, oral health changes, mental health summary, physical and mental health, physical health summary, vietnam, end-stage kidney disease, kidney transplant recipients, nutrition status

## Abstract

Background

Malnutrition is strongly associated with lower quality of life (QoL) and lower survival rates in patients with end-stage kidney disease. However, the impact of renal transplantation on nutrition factors and QoL is unclear. Therefore, this study aims to assess changes in QoL and investigate the relationships with nutrition factors among kidney transplant recipients (KTRs).

Materials and methods

A longitudinal study included 86 dialysis patients aged 18-65 years who underwent primary kidney transplantation (KTx) and were followed up for one year. Body weight, biochemical parameters, and QoL data were collected before transplantation (T0) and at six months (T6) and 12 months (T12) post-transplantation. Effect size (ES) was used to measure the impact of KTx on QoL and nutritional status from T0 to T12. The predictors of QoL were calculated with β-coefficients and p<0.05 in linear regression.

Results

The ES of transplantation on the QoL of KTRs was large, at 1.1 for health change, 0.9 for physical health, and moderate (0.7) for mental health (MH) over one year. Hemoglobin and malnourished were affected by KTx, with ES being 2.4 and 0.6, respectively. Linear regression showed that physical health was predicted by hemoglobin (β=0.12, p<0.01), phosphorus (β=7.82, p<0.05), and dose of mycophenolate mofetil (MMF) (β=-0.01, p<0.05). Mental health was predicted by obesity (β=-7.63, p<0.05), hemoglobin (β=0.11, p<0.05), and phosphorus (β=8.49, p<0.01). Health change was indicated by nutritional risk index (NRI) score (β=0.47, p<0.05), total cholesterol (β=3.39, p<0.01), and kidney function (β=0.15, p<0.05).

Conclusions

The transition from end-stage kidney disease to transplantation has positive impacts on QoL and nutrition markers. Nutritional status, kidney function, and the dose of mycophenolate mofetil are significant determinants of QoL in KTRs.

## Introduction

Chronic kidney disease (CKD) significantly impacts the daily lives of people undergoing dialysis, manifesting symptoms such as fatigue, sleeping disorders, and challenges in managing familial responsibilities. While dialysis temporarily alleviates some symptoms of CKD, it often results in decreased quality of life (QoL) due to frequent hospitalization and low survival rate [1,2]. Conversely, kidney transplantation (KTx) offers a transformative solution, as it enhances kidney function and overall health [3]. After transplantation, the quality of life (QoL) can be determined by objective factors such as the disease and its treatment and subjective factors such as the individual's satisfaction with life [4].

Prior studies, using tools such as the 36-Item Short-Form Health Survey (SF-36), have extensively analyzed recipients' QoL in the transition from hemodialysis or peritoneal dialysis to kidney transplantation and concluded that there was a significant improvement in the overall rating of QoL [5,6]. These studies predominantly reported enhancements in physical health components [7,8] and perceptions of overall health [2,8,9] following transplantation. However, long-term assessments reveal nuances, with some studies indicating sustained [6] or diminished [5]. QoL post-transplantation is partly attributable to factors such as post-transplantation CKD and psychosocial burdens [5,10,11].

Psychological factors, such as the fear of graft failure and adherence to medication, are known to have a significant influence on postoperative treatment outcomes [10,12]. Other studies from Asian countries also reported that adherence to immunosuppressant medicines [13], alongside sociocultural aspects such as illness representation, also significantly influences the QoL in kidney transplant recipients (KTRs) [3] and CKD patients [14]. Furthermore, nutritional status emerges as a critical determinant, with malnutrition commonly observed in CKD patients and strongly associated with diminished QoL [15]. A cohort study in 2022 identified malnutrition and reduced dietary fat intake as independent predictors of lower physical component summary (PCS) and mental component summary (MCS) within one year post-transplantation [1]. Another study showed that PCS and MCS increased by 0.74 and 0.36 points per 10 g/day increment of dietary protein intake, but after adjustment for bias and confounders, MCS showed no association [16]. Additionally, nutritional factors, such as diabetes, hemoglobin [1,5], albumin, lipids profile [6], and creatinine level [11], were associated with QoL over time. Despite the associations, the precise impact of nutritional changes on the QoL trajectory of KTRs remains insufficiently explored in the current literature.

To address this gap, we conducted a prospective study tracking KTRs before and after kidney transplantation over a year. The research included regular follow-ups to monitor changes in QoL and measured nutritional status with specific biomarkers. This approach provided the effects of kidney transplantation on the QoL of individuals transitioning from end-stage kidney disease to transplantation and examined the influence of nutritional status on QoL in KTRs.

## Materials and methods

Study design and participants

A prospective cohort study was conducted at a hospital in Vietnam from March 2021 to March 2023. To be included in the study, KTRs aged 18-65 years had undergone primary kidney transplantation, and they were followed up for one year after kidney transplantation. The participants were given an explanation of the study, reviewed the patient information sheet, and signed a written consent form before joining the study. Individuals were excluded from the study if they lacked screening data at 108 Military Central Hospital (108 MCH), Vietnam; experienced acute rejection within the first month after transplantation; or were diagnosed with cancer, liver cirrhosis, heart failure, mental disorders, or physical disabilities.

Data was initially collected from 106 participants. Missing data was identified through preliminary data screening, where each variable was checked for completeness. The participants with missing data on any of the variables of interest were excluded from the final analysis. This resulted in the inclusion of 86 participants who had complete data. In total, the study included 86 KTRs.

The study was approved by the Institutional Review Board of 108 MCH and Universiti Kebangsaan Malaysia issued approval 2021-1539/HDDD (29 March 2021) and JEP-2021-663 (15 October 2021), respectively.

Data collection procedures

The subjects' age, weight, etiology of end-stage kidney disease, time and type of renal replacement therapy before transplantation (T0), and donor source were obtained on registration day two weeks before the operation (T0). Patients' weight, blood parameters, and quality of life were recorded at baseline (T0), one day before discharge (within the first month post-transplantation {T1}), and during clinic visits six months (T6) and 12 months (T12) after kidney transplantation. After kidney transplantation, all the recipients were treated with triple immunosuppressive therapies, including tacrolimus (TAC), mycophenolate mofetil (MMF), and prednisolone. In the maintenance therapy, the target blood trough level of 7-10 ng/mL was recommended for TAC; the dose of MMF ranged from 1500 to 2000 mg/day, and the daily prednisolone dose was 5 mg/day.

Nutritional status

Body mass index (BMI) was categorized following classification for the Asia Pacific population: BMI>25 kg/m^2^ was obese, and BMI<18.5 kg/m^2^ was underweight. The nutritional risk index (NRI), calculated based on serum albumin level, body weight, and ideal body weight, was utilized to evaluate the risk of malnutrition for patients under 65 years old. Patients were categorized as having a moderate and severe risk of being malnourished if their NRI scores fell below 97.5 [17].

Clinical characteristics

Nutritional markers, such as serum hemoglobin, albumin, phosphorus, and magnesium, and biochemical parameters, including fasting serum glucose, serum triglycerides, uric acid, and serum creatine, were obtained from the hospital's laboratories. The tacrolimus level, MMF, and prednisolone doses were recorded. The estimated glomerular filtration rate (eGFR) was calculated using the Chronic Kidney Disease Epidemiology Collaboration creatinine equation.

Quality of life

Information on health-related QoL (HRQoL) was collected initially and every six months post-transplantation through interviews utilizing the 36-Item Short-Form Health Survey (SF-36). This survey comprised 36 questions covering several domains [18]: (i) physical functioning (PF), (ii) role limitations due to physical health or role physical (RP), (iii) bodily pain (BP), (iv) general health (GH), (v) social functioning (SF), (vi) role limitations due to emotional problems or role emotion (RE), (vii) vitality (VT), (viii) mental health (MH), and (ix) change in health (HC). The physical component summary (PCS) comprised PF, RP, BP, and GH, while the mental component summary (MCS) included SF, RE, VT, and MH. Higher scores indicated a better QoL. The SF-36 version 2.0 in Vietnamese underwent modification and validation in 2008. KTRs were asked to self-evaluate and complete the SF-36. Two researchers, each with a decade of survey experience, reviewed the forms to ensure they were complete. If any sections have missing information, the researchers interviewed the KTRs to complete the forms [19].

Statistical analysis

Multiple checks were performed during data entry and cleaning to ensure the accuracy and completeness of the dataset. Statistical analyses were performed using SPSS for Windows version 26.0 (IBM SPSS Statistics, Armonk, NY). The final dataset, comprising only those participants with complete data, was subjected to various statistical analyses. The comparison of outcome variables in nutritional markers and quality of life (QoL) was conducted between pre- and post-measurements within subjects utilizing paired t-tests. Effect sizes (ES) were computed as the mean change score of QoL in each domain between T12 and T0, divided by the standard deviation of the initial mean score in that domain [20]. ES were categorized as small (ES=0.20), moderate (ES=0.50), and large (ES=0.80) [9]. A positive effect size indicated an improvement in QoL, while a negative effect size indicated a deterioration. QoL scores associated with nutritional markers and biochemical parameters were determined via linear regression, providing β coefficients and a 95% confidence interval (CI).

## Results

**Figure 1:**
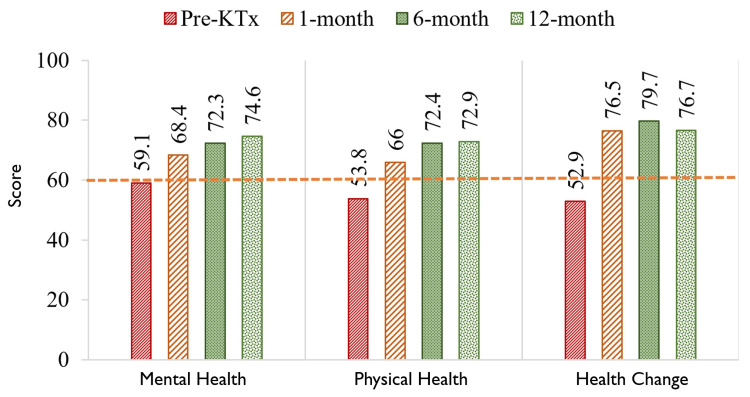
Changes in physical and mental health summary and the previous year's changes in the health of kidney transplant recipients The line references the quality of life score of Vietnamese people with kidney failure [21] KTx: kidney transplantation

The characteristics of kidney transplant recipients are listed in Table 1. The quality of life before transplantation was notably low, with mean scores of mental component summary, physical component summary, and health changes (HC) at 59, 54, and 53 points, respectively. The line cites the overall quality of life scores from Vietnamese patients [21]. Following kidney transplantation, there was significant improvement in SF-36 scores, with scores surpassing 60 points at the first follow-up (T1) and reaching 70 points at subsequent follow-ups (T6 and T12) (Figure 1).

**Table 1:** Characteristics of kidney transplant recipients SD: standard deviation

Variables	Total (n=86)	Male (n=63)	Female (n=23)
Age, mean in years±SD	40.7±11.8	40.7±12.0	40.8±11.4
Dialysis before transplantation, %	89.5	88.9	91.3
Etiology of transplantation, %			
Glomerulonephritis	79.1	82.5	69.6
Hypertension	8.1	6.3	13.0
Diabetic	5.8	7.9	0.0
Others (lupus, IgA nephrology)	7.0	3.2	17.4
Induction therapy, %			
Basiliximab	82.6	79.4	91.3
Antithymocyte globulin	8.1	9.5	4.3
No induction	9.3	11.1	4.3
Length of hospital stays, mean in days±SD	20.0±14.7	21.2±15.9	16.7±10.6
Hospitalization due to kidney transplantation, %	26.7	30.2	17.4

Impact of kidney transplantation on the quality of life in KTRs

Table 2 illustrates the effect of kidney transplantation on the QoL in KTRs. Notably, the previous year's health changes exhibited the highest ES of 1.06, followed by PCS and general health with 0.87 and 0.83, respectively. Other physical health domains showed moderate impact, with ES ranging from 0.6 to 0.7. Kidney transplantation moderately affected mental health components and the total MCS, with ES ranging from 0.5 to 0.7. Regarding nutritional status, weight and BMI decreased in the initial six months post-transplantation but exhibited significant improvements in the subsequent six months. Kidney transplantation also moderately affected the risk of malnutrition at the one-year follow-up. Moreover, kidney transplantation greatly impacted the anemia status in KTRs, with ES at T6 and T12 being 1.9 and 2.3, respectively.

**Table 2:** Effect size (ES) of transplantation on quality-of-life domains and nutritional status during follow-up The SF-36 score was calculated by value in post-transplantation minus T0; the effect size was calculated by Δ divided by SD of T0 *p<0.05 **p<0.01 ***p<0.001 BMI, body mass index; NRI, nutritional risk index; SD, standard deviation; Δ, mean change; SF-36, 36-Item Short-Form Health Survey; T0, before transplantation; T1, one month post-transplantation; T6, six months post-transplantation; T12, 12 months post-transplantation

SF-36 domains	Pretransplantation	Difference in T1 and T0		Difference in T6 and T0		Difference in T12 and T0	
	Mean±SD	Δ	ES	Δ	ES	Δ	ES
Health changes	52.9±22.5	23.5	1.05^***^	26.7	1.19^***^	23.8	1.06^***^
Physical components summary	53.8±21.8	12.2	0.56^***^	18.6	0.85^***^	19.0	0.87^***^
Physical functioning	66.4±24.0	9.5	0.40^**^	15.9	0.66^***^	14.9	0.62^***^
Role physical	42.4±42.9	7.6	0.18	23.8	0.56^***^	27.6	0.64^***^
Bodily pain	66.3±23.6	12.1	0.51^**^	16.9	0.72^***^	17.3	0.73^***^
General health	40.2±19.6	19.7	1.01^***^	17.6	0.90^***^	16.3	0.83^***^
Mental component summary	59.1±21.4	9.2	0.43^**^	13.1	0.61^***^	15.4	0.72^***^
Social functioning	61.5±20.8	7.0	0.33^*^	12.6	0.61^***^	11.8	0.56^***^
Role emotional	49.2±46.2	3.9	0.08	16.7	0.36^*^	25.2	0.55^***^
Vitality	59.0±19.6	15.5	0.79^***^	13.8	0.71^***^	14.5	0.74^***^
Mental health	66.9±15.8	10.7	0.67^***^	9.4	0.60^***^	10.3	0.65^***^
Nutritional status							
Weight, kg	55.3±8.7	-2.27	-0.26^***^	-1.23	-0.14^*^	1.66	0.19^*^
BMI, kg/m^2^	20.6±2.8	-0.83	-0.30^***^	-0.34	-0.12	0.51	0.19^*^
NRI, score	101.2±8.0	1.22	0.15	4.33	0.54^***^	4.56	0.57^***^
Hemoglobin	109.0±15.1	8.34	0.55^***^	29.3	1.94^***^	34.4	2.27^***^

Factors associated with the quality of life in KTRs

Table 3 summarizes the univariate analysis between nutritional markers and quality of life components. Hemoglobin and phosphorus levels were positively correlated with PCS and MCS scores, while obesity was negatively correlated with MCS. For HC score, being male, NRI, cholesterol level, and eGFR were associated factors.

**Table 3:** Factors influencing physical health, mental health, and the previous year's health changes BMI, body mass index; eGFR, estimated glomerular filtration rate; TAC, tacrolimus; MMF, mycophenolate mofetil; CI, confidence interval

Factors	Physical component summary score			Mental component summary score			Health changes score		
	β	95% CI	p	β	95% CI	p	β	95% CI	p
Age, years	0.11	-0.09, 0.31	0.27	0.12	-0.08, 0.31	0.25	0.02	-0.21, 0.25	0.86
Male	-1.54	-6.81, 3.74	0.57	-4.06	-9.27, 1.16	0.13	-7.56	-13.7, -1.41	0.02
Obesity	-4.29	-11.4, 2.81	0.24	-7.62	-14.6, -0.62	0.03	2.53	-5.85, 10.9	0.55
Underweight	0.52	-5.64, 6.67	0.87	-1.45	-7.56, 4.66	0.64	2.47	-4.77, 9.71	0.50
Risk of malnutrition	0.50	-8.59, 9.59	0.91	-4.76	-13.7, 4.24	0.30	4.31	-6.37, 15.0	0.43
Nutritional risk index, score	0.20	-0.16, 0.55	0.28	-0.13	-0.48, 0.22	0.46	0.47	0.06, 0.88	0.03
BMI, kg/m^2^	0.04	-0.73, 0.80	0.93	-0.43	-1.18, 0.33	0.27	0.74	-0.15, 1.64	0.10
Albumin, mg/L	0.61	-0.28, 1.50	0.18	-0.11	-0.99, 0.78	0.81	0.19	-0.86, 1.24	0.72
Hemoglobin, g/L	0.12	0.04, 0.21	<0.01	0.11	0.03, 0.19	0.01	0.10	0, 0.20	0.05
Phosphorus, mmol/L	7.82	2.11, 13.5	0.01	8.49	2.88, 14.1	<0.01	1.15	-5.58, 7.89	0.74
Magnesium, mmol/L	6.64	-9.98, 23.2	0.43	11.58	-5.07, 28.2	0.17	-6.89	-26.4, 12.7	0.49
Cholesterol, mmol/L	1.34	-0.50, 3.18	0.15	-0.38	-2.21, 1.45	0.68	3.39	1.26, 5.53	<0.01
Triglyceride, mmol/L	-1.45	-3.60, 0.70	0.19	-1.83	-3.96, 0.30	0.09	-0.76	-3.29, 1.78	0.56
Fasting glucose, mmol/L	0.03	-0.17, 0.22	0.78	0.03	-0.16, 0.23	0.73	-0.16	-0.39, 0.07	0.17
Uric acid, µmol/L	-0.02	-0.06, 0.01	0.21	-0.01	-0.05, 0.02	0.54	-0.02	-0.06, 0.02	0.37
Serum creatinine, µmol/L	0.01	-0.05, 0.06	0.83	0	-0.05, 0.05	0.99	-0.03	-0.09, 0.03	0.28
eGFR, mL/minute/1.73 m^2^	0.10	-0.01, 0.21	0.08	0.10	-0.01, 0.21	0.07	0.15	0.02, 0.28	0.03
TAC, ng/mL	-0.32	-1.00, 0.36	0.36	-0.23	-0.90, 0.45	0.51	-0.45	-1.25, 0.35	0.27
Dose of MMF, mg	-0.01	-0.02, 0	0.03	-0.01	-0.01, 0	0.05	0.01	0, 0.01	0.17
Corticoid, mg	0.04	-0.03, 0.12	0.24	0.06	-0.02, 0.13	0.12	0.02	-0.07, 0.10	0.74

The univariate analysis for the association of nutritional predictors with four physical health domains was presented in Table 4. Table 5 illustrates the association with four mental health domains. Phosphorus and hemoglobin levels showed associations with most QoL domains, with phosphorus levels being associated with six domains and hemoglobin levels with four domains. Both factors exhibited positive associations with bodily pain, physical functioning, and role emotion. Obesity demonstrated negative correlations with RP and RE, whereas NRI positively correlated with bodily pain. Recipient age at transplantation, albumin, and cholesterol had positive correlations with GH score. Regarding immunosuppressive therapy, the target troughs of tacrolimus and prednisolone did not correlate with any QoL domains. However, the maintenance dose of MMF showed a negative association with PCS, PF, RP, and RE.

**Table 4:** Factors influencing physical health components: regression analysis results *p<0.05 **p<0.01 NRI, nutritional risk index; BMI, body mass index; eGFR, estimated glomerular filtration rate; MMF, mycophenolate mofetil; CI, confidence interval

Factors	Physical functioning		Role physical		Bodily pain		General health	
	β	95% CI	β	95% CI	β	95% CI	β	95% CI
Age, years	0.1	-0.11, 0.31	0	-0.42, 0.42	0.09	-0.13, 0.32	0.25^*^	0.07, 0.43
Male	0.77	-4.79, 6.33	-5.31	-16.5, 5.91	0.98	-5.07, 7.03	-2.59	-7.50, 2.32
Obesity	2.4	-5.10, 9.89	-16.5^*^	-31.5, -1.49	0.27	-7.88, 8.43	-3.32	-9.94, 3.30
Underweight	0.61	-5.87, 7.10	0.9	-12.2, 14.0	3.69	-3.35, 10.7	-3.14	-8.86, 2.59
Risk of malnutrition	1.6	-7.97, 11.1	-6.39	-25.7, 12.9	2.87	-7.54, 13.3	3.91	-4.54, 12.4
NRI, score	0.17	-0.20, 0.55	-0.03	-0.79, 0.72	0.43^*^	0.03, 0.83	0.21	-0.12, 0.54
BMI, kg/m^2^	0.01	-0.79, 0.82	-0.29	-1.92, 1.34	0.52	-0.35, 1.40	-0.1	-0.82, 0.61
Albumin, mg/L	0.7	-0.23, 1.64	0.02	-1.88, 1.92	0.83	-0.18, 1.85	0.88^*^	0.05, 1.70
Hemoglobin, g/L	0.09^*^	0.01, 0.18	0.23^*^	0.05, 0.41	0.13^*^	0.03, 0.22	0.04	-0.04, 0.11
Phosphorus, mmol/L	8.03^*^	1.80, 14.2	10.97	-1.38, 23.3	7.99^*^	1.45, 14.5	4.3	-0.98, 9.58
Magnesium, mmol/L	7.83	-9.77, 25.4	11.15	-24.3, 46.6	6.89	-12.3, 26.1	0.68	-14.8, 16.2
Cholesterol, mmol/L	0.37	-1.58, 2.31	0.99	-2.95, 4.92	2.1	-0.01, 4.20	1.91^*^	0.20, 3.61
Triglyceride, mmol/L	-2.46^*^	-4.71, -0.21	-3.16	-7.74, 1.42	-0.95	-3.42, 1.52	0.77	-1.24, 2.78
Fasting glucose, mmol/L	0.02	-0.19, 0.22	0.01	-0.41, 0.42	0.01	-0.21, 0.23	0.08	-0.10, 0.26
Uric acid, µmol/L	-0.01	-0.05, 0.02	-0.05	-0.13, 0.02	-0.01	-0.05, 0.03	-0.01	-0.05, 0.02
Serum creatinine, µmol/L	0.04	-0.02, 0.09	0.01	-0.10, 0.12	-0.01	-0.07, 0.05	-0.02	-0.06, 0.03
eGFR, mL/minute/1.73 m^2^	-0.04	-0.16, 0.08	0.17	-0.07, 0.41	0.13	0, 0.26	0.15^**^	0.05, 0.26
Tacrolimus, ng/mL	-0.38	-1.09, 0.34	-0.48	-1.92, 0.97	-0.5	-1.29, 0.28	0.08	-0.56, 0.73
Dose of MMF, mg	-0.01^*^	-0.02, 0	-0.02^*^	-0.04, -0.01	0	-0.01, 0	0	-0.01, 0.01
Corticoid, mg	0.02	-0.06, 0.09	0.08	-0.07, 0.24	0.03	-0.05, 0.12	0.04	-0.03, 0.11

**Table 5:** Factors influencing mental health components: regression analysis results *p<0.05 **p<0.01 NRI, nutritional risk index; BMI, body mass index; eGFR, estimated glomerular filtration rate; MMF, mycophenolate mofetil; CI, confidence interval

Factors	Social functioning		Role emotional		Vitality		General mental health	
	β	95% CI	β	95% CI	β	95% CI	β	95% CI
Age, years	0.11	-0.12, 0.35	0.12	-0.29, 0.52	0.15	-0.03, 0.32	0.09	-0.08, 0.25
Male	-4.12	-10.4, 2.16	-5.66	-16.5, 5.22	-3.58	-8.24, 1.09	-2.87	-7.23, 1.49
Obesity	-7.53	-16.0, 0.92	-17.06	-31.6, -2.51	-2.38	-8.70, 3.94	-3.53	-9.41, 2.35
Underweight	0.77	-6.57, 8.12	-2.65	-15.3, 10.0	-1.89	-7.35, 3.57	-2.04	-7.13, 3.06
Risk of malnutrition	-3.28	-14.1, 7.55	-12.32	-31.0, 6.38	-2.16	-10.2, 5.91	-1.27	-8.80, 6.25
NRI, score	-0.21	-0.63, 0.22	-0.29	-1.02, 0.44	0.04	-0.28, 0.35	-0.07	-0.37, 0.22
BMI, kg/m^2^	-0.42	-1.33, 0.49	-0.78	-2.35, 0.80	-0.21	-0.89, 0.47	-0.3	-0.93, 0.33
Albumin, mg/L	-0.36	-1.42, 0.71	-0.24	-2.08, 1.60	0.23	-0.57, 1.02	-0.06	-0.80, 0.68
Hemoglobin, g/L	0.1	0, 0.20	0.22	0.05, 0.39	0.06	-0.02, 0.14	0.06	-0.01, 0.13
Phosphorus, mmol/L	10.3^**^	3.37, 17.2	12.3^*^	0.46, 24.2	5.21^*^	0.17, 10.2	6.09^*^	1.43, 10.8
Magnesium, mmol/L	18.07	-2.10, 38.2	17.16	-17.4, 51.7	6.83	-8.14, 21.8	4.25	-9.62, 18.1
Cholesterol, mmol/L	-0.73	-2.93, 1.48	-2.03	-5.83, 1.77	0.77	-0.87, 2.40	0.48	-1.05, 2.01
Triglyceride, mmol/L	-1.74	-4.30, 0.83	-4.69^*^	-9.11, -0.27	-0.9	-2.82, 1.02	0	-1.79, 1.79
Fasting glucose, mmol/L	0.06	-0.17, 0.29	0.14	-0.26, 0.54	-0.03	-0.20, 0.15	-0.04	-0.20, 0.12
Uric acid, µmol/L	0	-0.04, 0.04	-0.02	-0.09, 0.05	-0.02	-0.05, 0.01	0	-0.03, 0.03
Serum creatinine, µmol/L	-0.03	-0.09, 0.03	0.01	-0.09, 0.12	0.01	-0.04, 0.06	0.01	-0.04, 0.05
eGFR, mL/minute/1.73 m^2^	0.13	0, 0.27	0.16	-0.07, 0.39	0.07	-0.03, 0.17	0.05	-0.04, 0.15
Tacrolimus, ng/mL	-0.59	-1.40, 0.23	-0.55	-1.95, 0.85	0.13	-0.48, 0.74	0.1	-0.47, 0.66
Dose of MMF, mg	-0.01	-0.01, 0	-0.02^*^	-0.04, -0.01	0	-0.01, 0.01	0	-0.01, 0.01
Corticoid, mg	0.06	-0.03, 0.14	0.08	-0.07, 0.23	0.05	-0.02, 0.11	0.04	-0.02, 0.10

## Discussion

Impact of kidney transplantation on the quality of life in KTRs

The current study evaluated the QoL of 86 KTRs using the SF-36 questionnaire over a period of 12 months. Assessments were conducted before transplantation and at one, six, and 12 months post-operation. The results indicated a moderate-to-high improvement in SF-36 domain scores and nutritional markers, following the transition from CKD to kidney transplantation. Notably, QoL exhibited significant improvement after the six-month post-transplantation mark (T6), although no significant differences were observed at the T12 mark.

While short-term improvements in QoL were evident among KTRs, there was no observed change in the long term. This pattern aligns with previous findings, suggesting an improvement in domains of QoL following transplantation, albeit with variations across different countries. Some studies showed insignificant changes in QoL during late post-transplantation periods [2,7]. For instance, research in Poland and Norway demonstrated significant improvements in physical and mental health components shortly after transplantation but no significant changes in the long term [7,8]. However, Turkish KTRs did not exhibit significant changes in QoL during six months of follow-up from month 3 to month 9, except PF [2]. Comparisons with healthy groups revealed significantly lower QoL among KTRs within the first year post-transplantation in Turkey [2], while certain aspects of QoL were comparable to healthy individuals in Bangladesh [22]. Notably, there were no significant differences in QoL among patients undergoing hemodialysis, peritoneal dialysis, and kidney transplantation [23].

Furthermore, our study observed a significant increase in "self-evaluated last year's health change" or "overall health rating" (HC domain) of 24 points in the transition from CKD to kidney transplantation, with ES being 1.1. This increase was more pronounced compared to findings from Norway, with the overall health rating score improved from 58 to 68 points in pre- and post-kidney transplantation [8]. A study in the Netherlands illustrated a more significant effect size of kidney transplantation on HC score with an ES of over 2.0. The preoperative HC score of patients in the Dutch study was only 28.3 points, and it increased significantly to 66.3 points post-operation [9].

The impact of transplantation on QoL domains was positive. Transplantation had moderately positive effect sizes on MCS and four domains of mental health components and had great effect sizes on HC, PCS, and two domains of physical health components. An earlier study with a similar design also reported significant improvements in five out of nine domains of SF-36 from before to three months after kidney transplantation. Specifically, kidney transplantation greatly impacted HC, GH, and VT at the three-month follow-up while having a moderate impact on SF and RP [9]. Kidney transplantation had the most significant impact on physical components due to the recovery of immediate kidney functions, despite potential postoperative complications, such as stress and catabolism. Recipients typically experience resolution of these complications within 1-2 weeks post-surgery [24], allowing for discharge and subsequent outpatient monitoring.

Factors associated with the quality of life in KTRs

In our study, obesity was negatively correlated with certain aspects of QoL, specifically RF, RE, and MCS scores. This result diverged from previous research, which indicated that malnutrition, as measured by Mini Nutritional Assessment, was associated with PCS but not significant with MCS [23]. However, other demographic factors in our study did not show any significant association with QoL domains. These results contrasted with evidence indicating that PCS was negatively associated with age, sex, education status, and income [25].

Existing literature suggested that QoL in KTRs was influenced by factors such as anemia, nutritional status, sleep disorders, and kidney function [25]. However, our study did not find evidence supporting the hypothesis that being underweight (BMI<18.5) and at high risk of malnourishment (NRI<97.5) were independent predictors for QoL. Instead, NRI, hemoglobin, phosphorus, and total cholesterol emerged as predictors for the SF-36 score. In our experience, when we divided the recipients into these categories, the number of malnourished recipients was small and not large enough to produce statistically significant results. Despite this, the estimated prevalence of malnutrition among KTRs underscored the importance of ongoing vigilance for potential malnutrition risks post-transplantation [23].

The relationship between immunosuppressive therapy post-kidney transplantation and QoL is complex and multifaceted, involving physical, psychological, and social factors. Pharmacological side effects from medications such as corticosteroids, calcineurin inhibitors (CNIs), and mammalian target of rapamycin (mTOR) inhibitors often include hypertension, gaining weight, diabetes, hyperlipidemia, and increased susceptibility to infections. These adverse effects can cause physical discomfort, reduced mobility, and psychological stress, detrimentally affecting QoL [4,26]. The chronic nature of immunosuppressive therapy requires lifelong adherence, which can be psychological challenges such as depression and anxiety, possibly stemming from concerns about health status or returning to dialysis [10,12]. Additionally, immunosuppressive therapy can contribute to metabolic syndrome, characterized by insulin resistance, obesity, dyslipidemia, and hypertension. These conditions not only increase the risk of cardiovascular diseases but also reduce the ability to perform daily activities, impairing overall well-being [27].

In our study conducted at 108 MCH, kidney transplant recipients were treated with a triple medication regimen consisting of MMF, TAC, and prednisolone. We discovered that higher doses of MMF were negatively correlated with PCS, PF, RF, and RE. Although higher doses of TAC showed a nonsignificant decreasing trend in all SF-36 domains, lower doses of TAC were linked to fewer side effects and a positive association with the PCS score [4,28]. Furthermore, reducing the MMF dose appeared to mitigate gastrointestinal and hematological adverse effects, thereby potentially improving the recipients' QoL [26].

Regarding mental health components, our study revealed that kidney transplantation had a moderate impact on improving MCS. While some studies indicate that treatment therapy and duration during 12 months of follow-up had minor effects on MCS scores [28], others link higher MCS to reduced hospitalization rates and lower incidence of complications such as new-onset diabetes and adverse effects of immunosuppression therapy [4]. Notably, a two-year randomized clinical trial reported that discontinuing cyclosporine led to reduced fatigue and improved vitality, suggesting that modifying immunosuppressive regimens could enhance QoL outcomes [29]. Furthermore, visible side effects such as weight gain or changes in appearance can impact self-esteem and social interactions, further diminishing QoL. Addressing these side effects, improving medication adherence, and offering comprehensive patient support can help mitigate the negative impacts on QoL.

Strengths and limitations

Kidney transplantation's benefits and complications often evolve over several years, and a longer follow-up period is essential to observe these changes. Our study's short follow-up duration limited our findings to primarily reflect the early postoperative phase, potentially overlooking late-stage complications or long-term improvements. Another limitation was that the absence of the evaluation of the Vietnamese version of the Kidney Disease and Quality of Life (KDQOL-36) questionnaire significantly affected the study's comprehensiveness [21]. Without this tool, we could not assess kidney transplantation's ES on these kidney-specific domains, including the burden of kidney disease, symptoms and problems of kidney disease, and effect of kidney disease on daily life. Future research should prioritize validating the KDQOL-36 questionnaire to ensure more reliable and comprehensive findings. Despite the limitations, the study's longitudinal design and the meticulous tracking of QoL variations pre- and post-kidney transplantation over a year were significant strengths. This approach provided valuable insights into the immediate effects of kidney transplantation and allowed for the observation of treatment, lifestyle modifications, and disease progression impacts on individual QoL domains. By capturing these multifaceted influences, the study highlighted the dynamic nature of QoL changes and their correlation with nutritional status over time. Moreover, understanding the impact of kidney transplantation on mental health issues was crucial for devising appropriate KTR management strategies. By identifying these mental health components, the study provided a foundation for developing integrated care approaches, including nutrition, psychology, and clinical therapy to enhance QoL in KTRs.

## Conclusions

In summary, this study offers valuable insights into the effects of kidney transplantation on the QoL of individuals transitioning from end-stage kidney disease to KTRs. It highlights the significance of the nutritional risk index and various nutrition biomarkers on QoL in KTRs. These findings underscore the need for an appropriate and tailored management protocol for kidney transplant recipients, according to individualized trajectories of their planned processes. Further prospective studies with longer follow-up times are warranted to elucidate the multifaceted elements influencing kidney disease-specific QoL, mental health issues, and nutritional determinants in KTRs. This would help develop effective interventions to enhance the KTRs' long-term well-being. Additionally, addressing limitations such as extending follow-up periods and validating culturally appropriate QoL assessment tools such as the KDQOL-36 for Vietnamese KTRs are crucial. These efforts will offer a more comprehensive and accurate understanding of the long-term impacts of kidney transplantation and inform the creation of more effective, personalized care strategies to improve KTRs' QoL.
